# Exploring the nature of perceived treatment burden: a study to compare treatment burden measures in adults with cystic fibrosis

**DOI:** 10.3310/nihropenres.13260.1

**Published:** 2022-05-09

**Authors:** Rana Altabee, Siobhan B. Carr, Janice Abbott, Rory Cameron, Daniel Office, Jessie Matthews, Nicholas Simmonds, Rebecca Cosgriff, David Turner, Jennifer Whitty

**Affiliations:** 1 Health Economics Group, Norwich Medical School, University of East Anglia, Norwich, NR4 7TJ, UK; 2 College of Applied Medical Sciences, King Saud bin Abdulaziz University for Health Sciences, Jeddah, 22384, Saudi Arabia; 3 Department of Paediatric Respiratory Medicine, Royal Brompton & Harefield NHS Trust, London, SW3 6NP, UK; 4 National Heart and Lung Institute, Imperial College London, London, SW7 2BX, UK; 5 School of Psychology, University of Central Lancashire, Preston, PR1 2HE, UK; 6 National Institute for Health Research (NIHR) Applied Research Collaboration (ARC) East of England (EoE), Cambridge, CB2 8AH, UK; 7 Adult Cystic Fibrosis Centre, Royal Brompton & Harefield NHS Trust, London, SW3 6NP, UK; 8 Cystic Fibrosis Trust, London, EC3N 1RE, UK

**Keywords:** cystic fibrosis, treatment burden, patient-reported outcomes

## Abstract

**Background:**

Despite the importance of reducing treatment burden for people with cystic fibrosis (CF), it has not been fully understood as a concept. This study aims to quantify the treatment burden perceived by CF adults and explore the association between different validated treatment burden measures.

**Methods:**

This is a cross-sectional observational study of CF adults attending a single large UK adult center. Participants completed an online survey that contained three different treatment burden scales; CF Questionnaire-Revised (CFQ-R) subscale, CF Quality of Life (CFQoL) subscale, and the generic multimorbidity treatment burden questionnaire (MTBQ).

**Results:**

Among 101 participants, the median reported treatment burden by the CFQ-R subscale was 55.5 (IQR 33.3 – 66.6), the CFQoL subscale was 66.6 (IQR 46.6 – 86.6), and the MTBQ reversed global score was 84.6 (IQR 73.1 – 92.3). No correlation was found between respondents’ demographic or clinical variables and treatment burden measured via any of the three measures. All treatment burden measures showed correlations against each other. More treatments were associated with high treatment burden as measured by the CFQ-R, CFQoL subscales, and the MTBQ. However, longer treatment time and more complex treatment plans were correlated with high treatment burden as measured by the CFQ-R and CFQoL subscales, but not with the MTBQ.

**Conclusions:**

Treatment burden is a substantial issue in CF. Currently, the only available way to evaluate it is with the CF-specific quality of life measure treatment burden subscales (CFQ-R and CFQoL); both indicated that treatment burden increases with more treatments, longer treatment time, and more complex treatments.

## Plain English summary

People with cystic fibrosis (CF) are required to adhere to a very complicated and exhausting treatment plan daily. This can result in the patients experiencing excessive burden from the high healthcare workload needed to maintain their health. Therefore, it is important to look for ways to reduce their treatment burden. Treatment burden is not yet a fully understood concept in CF and different people could perceive treatment burden differently. This study aims to measure the treatment burden perceived by adults with CF and investigate the performance of three different treatment burden questionnaires.

This single survey study targeted 101 adults with CF in a large UK adult center. Participants completed an online survey that contained three different treatment burden measures; two short (three questions each) CF-specific questionnaires, and one generic (13 questions) questionnaire that captures treatment burden in people with long-term conditions.

The participants' responses suggested that people with CF have a high level of treatment burden according to the two CF-specific questionnaires, but not with the generic one. An important finding was that the severity of the participants' disease (based on their lung function) did not appear to affect the reported treatment burden. We found that age, gender, body mass index, and the number of intravenous antibiotic courses received in the previous year did not affect the reported treatment burden. Treatment burden was higher in people who had more treatment, spent more time doing their daily treatments, and received more complex treatment plans.

Currently, the CF-specific treatment burden questionnaires appear to be the most appropriate way to capture treatment burden in adults with CF. We believe that including treatment burden evaluation in the process of treatment decision-making is important as it takes into perspective the CF patient’s point of view about their treatment plan.

## Introduction

Survival for people with cystic fibrosis (pwCF) has increased over the years. According to the UK CF Registry in 2020, the median predicted survival for pwCF born today is 50.6 years ^1^. This is due to multiple treatments and prevention therapies ^2^. To maintain the health of pwCF, a complicated treatment regimen is needed daily. This includes nutritional management, inhalation therapies, sputum clearance using chest physiotherapy, antibiotic therapy, and cystic fibrosis transmembrane conductance regulator (CFTR) modulators ^3^. It is estimated that adults with CF require two to three hours daily to complete their treatments resulting in a high burden for them or their caregivers ^3^.

Treatment burden is a result of healthcare workload experienced by patients with chronic conditions and their caregivers that affects their lives and well-being ^4^. This workload involves patient’s time and effort to complete the treatment in addition to other self-care tasks such as monitoring the condition, nutritional management, and exercise ^4^. High treatment burden may be associated with low quality of life, low adherence to treatment, and inefficient use of medical resources if people cannot adhere to treatment plans ^5^. Treatment burden is considered an important issue and ranked as the top research question for pwCF, caregivers, and clinicians according to James Lind Alliance's top CF questions list ^6^.

Treatment burden could be induced by the treatment type, amount, frequency, duration, learning and remembering how to manage treatments, sterilization of medical equipment, or the constant monitoring of the condition at home ^7, 8^. Also, the complexity of administration for some treatments could contribute to increasing the perceived treatment burden ^7^. Nonetheless, these factors may not necessarily equate to the burden perceived by a person with CF as they are objective aspects of treatment burden. Each person perceives treatment burden differently; it is subjective to the person with CF ^9^.

Currently, the only available instruments to capture perceived treatment burden for pwCF are the treatment burden subscales of the CF-specific quality of life measures; the CF questionnaire revised (CFQ-R) and the CF quality of life (CFQoL) instruments ^10, 11^. Both subscales cover some concepts of treatment burden such as time spent on treatment, difficulties caused by the treatments, and interference with life and happiness. However, these subscales only focus on specific aspects of treatment burden and neglect other areas such as the burden of financial and managerial requirements associated with treatment. Despite the lack of complete CF-specific instruments, there are generic treatment burden instruments that are used with different conditions. These instruments include the Multimorbidity Treatment Burden Questionnaire (MTBQ) developed for a UK population and the Treatment Burden Questionnaire (TBQ) ^12, 13^. However, these generic instruments have not been evaluated in pwCF. There are no available studies that have compared the performance of different treatment burden instruments ^8^.

In this study, we aimed to quantify perceived treatment burden in CF adults. Moreover, we hypothesized that high treatment burden is associated with more treatments, longer treatment time, and more complex treatment; therefore, we assessed the performance of the two available treatment burden subscales in CF-specific quality of life measures (CFQ-R and CFQoL) and the generic treatment burden measure (MTBQ) in capturing perceived treatment burden and their correlation with each other. A secondary aim was to explore the relationship between perceived treatment burden and disease severity.

## Methods

### Study population

This cross-sectional, observational survey study recruited CF adults aged 18 years or older between July and October 2020 from an adult CF center in London, UK. The study was undertaken as part of a larger study investigating Evidence-based VALUation of patient outcomes in Cystic Fibrosis (the VALU-CF study). The participants completed an online survey that contained treatment burden subscales from the CFQ-R, CFQoL and the MTBQ ^14^. Also, the VALU-CF study collected discrete choice experiment (DCE) in the survey and time trade-off through interviews, which will be undertaken by a different study. The VALU-CF sample was determined using a rule of thumb calculation to estimate the sample needed for the DCE element of the survey ^15^. Based on the calculation, and allowing for a 15% drop out rate, the sample size was set at 108 patients.

The sample were recruited through telephone and email to complete an online or PDF survey. A £10 financial incentive was offered to the participants for completing the survey. Ethical approval was received for the VALU-CF study (REC: 19/YH/0423). An online written informed consent was obtained from the participants prior to completing the survey for participation and Registry data linkage.

### Measures

***Demographic and clinical variables.*** Demographic (age, gender, ethnicity, education level, marital status, employment status) and clinical (height, weight, body mass index “BMI”, percent predicted forced expiratory volume in 1 second “ppFEV1”, number of IV antibiotic courses received in the year prior to enrollment) variables for the participants were collected from the survey, the closest encounter data within the UK CF Registry and their CF center’s medical record.

***Treatment descriptors.*** The participants were asked to provide information related to their CF treatment including how much time they spent on inhaled therapies, chest physiotherapy, and other treatments. Based on these questions, total treatment time was calculated. Furthermore, the number of treatments, their types, (inhalers, nebulizers, and chest physiotherapy) and frequencies were collected from the Registry.

To assess the difficulty of doing these treatments, a treatment complexity score (TCS) was estimated for each participant. This measure was developed by Sawicki *et al*. ^7^ to give each CF treatment a score, ranging between 1 and 3, based on its frequency, administration time, and method. The scores for all treatments a participant was taking were added together to give a single TCS score (range 0 – 73) ^7^. A high TCS score suggests high treatment complexity. The TCS scoring in this study was based on the Sawicki *et al*. ^7^ study with some additions/modification, generated by an expert group including pwCF ( Table 1).

**Table 1.** The modified version of Sawicki *et al.* ^7^’s treatment complexity score table.

TCS Score = 1 point	TCS Score = 2 points	TCS Score = 3 points
Acid blockers	Antibiotics (nebulized OD)	Antifungals (inhaled) *
Analgesics	DNase (OD / OR)	Antibiotics (nebulized (BD/TDS)
Angiotensin receptor agonists *	Hypertonic saline (OD)	DNase (BD) *
Antibiotics (inhaled DPI) *	Pancreatic enzymes	Hypertonic saline (BD) *
Anticoagulants *	CFTR modulator *	Mannitol (DPI)
Antidepressants		Insulin
Antiemetics *		Colistin (nebulized) *
Antiepileptic *		Oxygen
Antifungals (oral) *		Airway clearance
Antihistamines *		Noninvasive ventilation *
Anti-inflammatories *		
Antiviral *		
Beta blocker *		
Bisphosphonates *		
Bronchodilators (inhaled)		
Bronchodilators (oral)		
Chronic oral antibiotics		
Corticosteroids (inhaled)		
Corticosteroids (inhaled) + LABA		
Corticosteroids (oral)		
Diuretics		
Immunosuppressants (oral) *		
Tranexamic acid 1 gm (TDS, PRN) *		
Metformin *		
Migraine prophylaxis *		
Minerals (oral)		
Nasal rinse/ spray *		
Prophylactic antibiotics (oral)		
Ropinirole *		
Statin *		
Tamoxifen *		
Vitamins (oral)		
Gastrointestinal medicines *		

* The newly added treatments to Sawicki *et al.* ^7^’s original version – none of the assigned treatments from the original version were moved to different categories or removed from the scale. **Abbreviations:** TCS = treatment complexity score, DPI = dry powder inhaler, LABA = long-acting beta agonist, TDS = three times a day, PRN = as required, OD = once a day, OR = other regimen, BD = twice a day.

***Treatment burden.*** Treatment burden was assessed using the CF-specific quality of life subscales from the CFQ-R (adult version), and the CFQoL, in addition to the generic treatment burden measure the MTBQ. Table 2 describes the treatment burden instruments used in this study, their development, validation and their items. Each of the treatment burden subscales in the CFQ-R and the CFQoL has three items that are scored on a 0 to 100-point scale; high scores indicate low perceived treatment burden ^10, 11^. The MTBQ is a generic treatment burden instrument that was developed in older adults with chronic conditions and consists of 13 items that capture treatment burden in people with multimorbidity ^12^. The MTBQ items generate a global score that ranges from 0 to 100, high scores indicate high perceived treatment burden ^12^.

**Table 2.** Description of the treatment burden measures used in this study.

Questionnaire	Development and validation	Type of instrument	Items related to treatment burden
**CFQ-R** *“Treatment burden” * *domain*	Developed by Henry *et al*. ^17^ and revised and validated by *Quittner* *et al.* ^11^ for CF.	A treatment burden subscale from a CF-specific quality of life measure.	Over the last two weeks, to what extent do your treatments make your daily life more difficult?
			Over the last two weeks, how much time do you currently spend each day on your treatments?
			Over the last two weeks, how difficult is it for you to do your treatments (including medications) each day?
**CFQoL** *“Treatment issues” * *domain*	Developed and validated by *Gee * *et al. ^10^* for CF.	A treatment burden subscale from a CF-specific quality of life measure.	Over the last two weeks, I have found my treatments (physio, enzymes etc.) very time consuming.
			During the last two weeks, my treatments have interfered with other things that I have wanted to do.
			Over the last two weeks, I have found that my treatments have interfered with my enjoyment of life.
**MTBQ**	Developed and validated by *Duncan et al.* ^12^ for patients with multimorbidity.	A generic treatment burden measure.	Taking lots of medications
			Remembering how and when to take medication
			Paying for prescriptions, over the counter medication or equipment
			Collecting prescription medication
			Monitoring your medical conditions (e.g. checking your blood sugar, monitoring your symptoms etc.)
			Arranging appointments with health professionals
			Seeing lots of different health professionals
			Attending appointments with health professionals (e.g. getting time off work, arranging transport etc.)
			Getting health care in the evenings and at weekends
			Getting help from community services (e.g. physiotherapy, district nurses etc.)
			Obtaining clear and up-to-date information about your condition
			Making recommended lifestyle changes (e.g. diet and exercise etc.)
			Having to rely on help from family and friends

### Statistical analysis

SPSS version 25 (IBM SPSS Statistics, RRID:SCR_016479) was used for data analysis. Descriptive statistics were derived for demographic and clinical variables, treatment descriptors (number of treatments, total treatment time, and TCS), and the treatment burden instruments. The MTBQ global score was reversed to ease the comparison with the CFQ-R and CFQoL (so that in all instruments; a high score represented low treatment burden). Shapiro-Wilk tests were carried out to determine normality of the data distribution for all variables. To explore how treatment burden differed according to disease severity, participants were divided into two disease severity groups based on their ppFEV1; mild: ≥70%, and moderate to severe: ≤69%.

Descriptive and inferential statistical analysis were employed to assess the three treatment burden instruments. First, a descriptive analysis was conducted on the three treatment burden instruments based on the disease severity groups. Then, to determine the nature of relationship between disease severity and treatment burden, we assessed the difference of the treatment burden reported by each of the three instruments between the disease severity groups. Due to skewedness of the treatment burden data, a Mann-Whitney test was used to determine the significance of the difference.

The associations between the three treatment burden instruments were investigated using Spearman’s Rank correlation test due to the lack of normality in the data. This helped in assessing the relationship between the treatment burden captured by the three instruments. A correlation was considered significant if the p-value was less than 0.05. A correlation coefficient was considered strong if it was higher than 0.7, moderate strength if it was between 0.7 and 0.3, and weak correlation if it was less than 0.3 ^16^. Furthermore, Spearman’s Rank test was conducted for each of the treatment burden instruments and demographic, clinical, and treatment descriptors (number of treatments, TCS, and total time of treatments) variables to assess the nature of their relationship.

## Results

### Descriptive statistics

The sample included 103 participants out of 276 invitations sent (response rate 37%), and two were excluded due to unavailability of their clinical and demographic data; therefore, 101 participants were included in the final analysis. Table 3 illustrates the demographic and clinical data. Table 4 shows detailed descriptive statistics of the treatments the participants were receiving at the time the study was conducted.

**Table 3.** Demographic and clinical variables for the sample.

Age in years (n=101)		Height in cm (n=101)	
Mean (SD)	35.7 (11.4)	Mean (SD)	168 (9.6)
Median (IQR)	34 (27 – 44)	Median (IQR)	168 (160.5 – 176.5)
Range	18 – 75	Range	143 – 187
Gender, n (%), (n=101)		Weight in kg (n=101)	
Female	52 (51.5%)	Mean (SD)	64.7 (13)
Male	49 (48.5%)	Median (IQR)	63 (54.6 – 73.5)
		Range	40.3 – 108
Ethnicity, n (%), (n=101)		BMI (n=101)	
White	98 (97%)	Mean (SD)	22.7 (3.1)
Other	3 (3%)	Median (IQR)	22.3 (20.8 – 24.4)
		Range	15.7 – 39.7
Education level, n (%), (n=101)		ppFEV1 (n=99) *	
University	50 (49.5%)	Mean (SD)	69.4 (22.2)
College	27 (26.7%)	Median (IQR)	69.1 (52.8 – 83)
High school	9 (8.9%)	Range	25.1 – 123.6
Less than high school	2 (2%)		
Not known	13 (12.9%)		
Marital status, n (%), (n=101)		FEV1 in liter (n=98) **	
Single	40 (39.6%)	Mean (SD)	2.47 (1.04)
Married	39 (38.6%)	Median (IQR)	2.31 (1.65 – 3)
Long-term partner	18 (17.8%)	Range	0.91 – 7
Divorced	2 (2%)		
Separated	1 (1%)		
Not known	1 (1%)		
Employment status, n (%), (n=101)		Number of IV antibiotic courses last year (n=101)	
Full-time	52 (51%)	Mean (SD)	0.62 (1.20)
Part-time	21 (20.6%)	Median (IQR)	0 (0 – 1)
Student	12 (11.8%)	Range	0 – 5
Unemployed	8 (7.88%)	Number of IV antibiotic days last year (n=101)	
Homemaker	3 (2.9%)	Mean (SD)	10.2 (22.1)
Retired	1 (1%)	Median (IQR)	0 (0 – 14)
Disabled	1 (1%)	Range	0 – 124
Not known	4 (3.9%)	CFRD, n (%), (n=101)	
		CFRD	29 (28.7%)

* Two participants did not have a reported ppFEV1. ** Three participants did not have a reported FEV1 in liters. **Abbreviations:** BMI = body mass index, ppFEV1 = percent predicted of forced expired volume in one second, CFRD = cystic fibrosis related diabetes.

**Table 4.** Descriptive statistics for the received treatments at the time of the study.

	Total (n=101)
Number of treatments	
Mean (SD)	13.2 (4.8)
Median (IQR)	13 (11 –16)
Range	0 – 31
Number of inhalers	
Mean (SD)	2 (1.2)
Median (IQR)	2 (1 – 3)
Range	0 – 5
Number of nebulizers	
Mean (SD)	2.3 (1.1)
Median (IQR)	2 (2 – 3)
Range	0 – 5
Number of chest physiotherapies	
Mean (SD)	1.5 (0.6)
Median (IQR)	1 (1 – 2)
Range	0 – 3
Type of primary chest physiotherapy, n (%)	
Autogenic drainage	36 (35.6%)
Active cycle breathing techniques	21 (20.8%)
Oscillating PEP	21 (20.8%)
Other	13 (12.9%)
None	10 (9.9%)
Inhaled medication time in minutes (min/day)	
Mean (SD)	42.6 (38.2)
Median (IQR)	30 (20 – 60)
Range	0 – 180
Chest physiotherapy time in minutes (min/day)	
Mean (SD)	36.3 (31.8)
Median (IQR)	30 (17.5 – 50)
Range	0 – 180
Other treatments time in minutes (min/day)	
Mean (SD)	12 (20)
Median (IQR)	5 (0 - 15)
Range	0 – 120
Total treatment time in minutes (min/day)	
Mean (SD)	91 (70.7)
Median (IQR)	80 (45 – 108.5)
Range	0 – 420
TCS (treatment complexity score)	
Mean (SD)	22 (7.4)
Median (IQR)	23 (18 – 27)
Range	0 – 40
CFTR Modulators, n (%),	
On CFTR modulator	66 (65.3%)
On Elexacaftor/tezacaftor/ivacaftor (Trikafta/Kaftrio ^©^)	33 (32.7%)

### Treatment burden and disease severity

Table 5 shows the treatment descriptors, and the treatment burden instruments descriptive statistics across the whole sample and the ppFEV1 disease severity groups. All disease severity groups had more than half of their participants receiving CFTR modulators (mild group; 61% and moderate to severe group; 70%). Half of those on CFTR modulators were taking elexacaftor/tezacaftor/ivacaftor (Trikafta/Kaftrio ^©^) in both disease severity groups. There were no statistically significant differences between disease severity groups for the CFQ-R subscale (U = 980.00, z = -1.73, p = 0.08), the CFQoL subscale (U = 1170.50, z = -0.38, p = 0.70), or the MTBQ reversed global score (U = 1028.00, z = -1.38, p = 0.16).

**Table 5.** Treatment descriptors and treatment burden instruments descriptive statistics based on disease severity groups.

	Mild severity (n=49)	Moderate to severe severity (n=50)	Total (n=99) *
Number of treatments			
Mean (SD)	11.3(4.1)	15 (4.9)	13.2 (4.8)
Median (IQR)	12 (9 – 13.5)	15 (13 – 17)	13 (11 – 16)
Range	2 – 20	0 – 31	0 – 31
TCS			
Mean (SD)	19 (6.4)	24.8 (7.3)	22 (7.5)
Median (IQR)	19 (14.5 – 24)	24 (21 – 29.5)	23 (18 – 27)
Range	5 – 32	0 – 40	0 – 40
Total treatment time (min/day)			
Mean (SD)	78.3 (45.1)	106 (87.5)	92.2 (70.8)
Median (IQR)	80 (45 – 100)	90 (50 – 125)	85 (50 – 110)
Range	0 – 190	0 – 420	0 - 420
CFQ-R ‘treatment burden” domain			
Mean (SD)	57.3 (22.1)	49.7 (23)	53.5 (22.7)
Median (IQR)	55.5 (44.4 – 72.2)	50 (33.3 – 66.6)	55.5 (33.3 – 66.6)
Range	11.1 – 100	11.1 – 100	11.1 – 100
CFQoL ‘treatment issues” domain			
Mean (SD)	64.7 (26.2)	63.3 (25.4)	64 (25.7)
Median (IQR)	73.3 (53.3 – 86.6)	66.6 (46.6 – 86.6)	66.6 (46.6 – 86.6)
Range	0 – 100	0 – 100	0 – 100
MTBQ reversed global score			
Mean (SD)	83.1 (13.6)	79.5 (14.1)	81.3 (14)
Median (IQR)	84.6 (74 – 94.2)	83.6 (69.2 – 90.3)	84.6 (73.1 – 92.3)
Range	46.1 – 100	42.3 – 100	42.3 – 100

* Two participants were not included due to the unavailability of their ppFEV1 data. **Abbreviations**: TCS = treatment complexity score.

### Correlation between the treatment burden instruments

**Figure 1.**
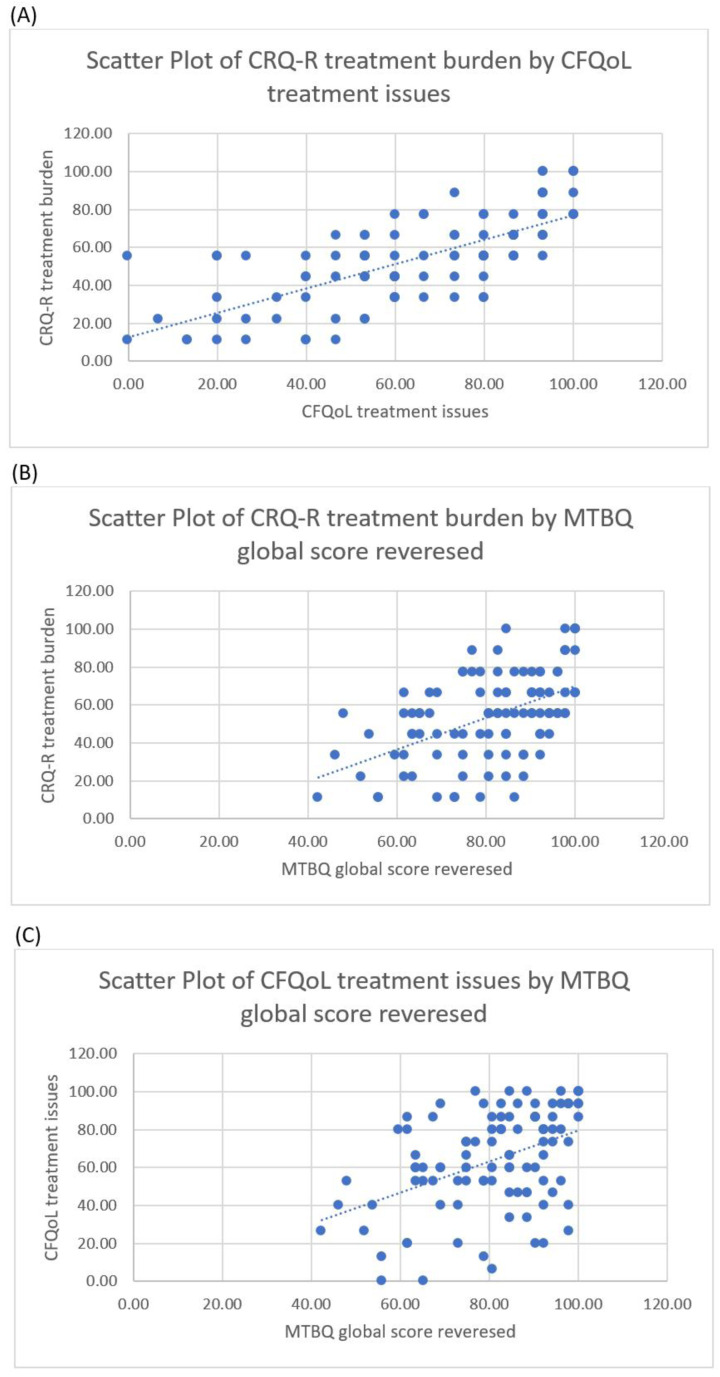
Scatter plots for the correlations between the three treatment burden instruments. ( **A**) the correlation between the CFQoL and the CFQ-R treatment burden subscales. ( **B**) the correlation between the CFQ-R treatment burden subscale and the MTBQ. ( **C**) the correlation between the CFQoL treatment burden subscale and the MTBQ.

Across the whole sample, statistically significant strong to moderate positive correlations were observed between treatment burden measured by the CFQ-R and CFQoL subscales (r _s _= 0.727, p <0.001), between the CFQ-R subscale and the MTBQ reversed score (r _s _= 0.511, p <0.001), and between the CFQoL subscale and the MTBQ (r _s _= 0.433, p <0.001). These correlations indicate that low observed treatment burden in any of the three instruments is associated with low treatment burden reported by the other instruments. Figure 1 shows the scatter plots of the correlation between the three treatment burden instruments.

### Correlation between the treatment burden instruments and demographic, clinical, and treatment descriptors variables

Age, gender, BMI, and the number of IV antibiotic courses received last year demonstrated no association with perceived treatment burden measured by any of the three instruments. Treatment burden measured by CFQ-R (r _s _= -0.424, p <0.01), CFQoL (r _s _= -0.305, p <0.01), and the MTBQ reversed score (r _s _= -0.220, p = 0.02) revealed significant moderate to weak negative correlations with the number of treatments, indicating that treatment burden may increase with more daily treatments. Among the treatment types, treatment burden measured by the CFQ-R demonstrated significant moderate associations with the number of inhaled therapies (r _s_= -0.324, p <0.01) and the number of nebulized therapies (r _s _= -0.363, p <0.01). Treatment burden measured by the CFQoL illustrated a significant weak correlation with the number of chest physiotherapy treatments (r _s _= -0.250, p = 0.01). The MTBQ reversed score showed a significant weak correlation with the number of inhaled therapies (r _s _= -0.261, p <0.01).

Treatment burden measured by the CFQ-R and CFQoL subscales showed significant negative moderate associations with TCS (r _s _= -0.428, p <0.01 and r _s _= -0.309, p <0.01, respectively). This indicates that high treatment complexity may associate with high perceived treatment burden. The MTBQ reversed score did not demonstrate any association with TCS (r _s _= -0.126, p = 0.12).

Total treatment time demonstrated significantly moderate to weak negative correlations with both CFQ-R and CFQoL treatment burden subscales with r _s _= -0.352, p <0.01 and r _s _= -0.246, p = 0.01, respectively. These results suggest that long treatment time could be associated with high perceived treatment burden. The MTBQ reversed score did not show any association with total treatment time (r _s _= -0.076, p = 0.45). Both CFQ-R and CFQoL demonstrated significantly moderate to weak negative correlations with inhaled medication time, (r _s _= -0.386, p <0.01 and r _s _= -0.243, p = 0.01, respectively). Only treatment burden measured by CFQ-R showed a significant weak negative correlation with chest physiotherapy time (r _s _= -0.210, p = 0.03). These findings indicate that long inhaled therapies and/or chest physiotherapy time might be associated with high perceived treatment burden.

## Discussion

A high degree of treatment burden was reported by pwCF, as measured by the CFQ-R and CFQoL. The MTBQ showed a slightly lower level of treatment burden compared to the other two subscales. There was no association observed between perceived treatment burden and the demographic and clinical variables; in addition to no significant difference in treatment burden between the disease severity groups categorized by lung function.

This study also aimed to assess the performance of the three treatment burden instruments (CFQ-R, CFQoL, MTBQ) and their correlation with each other. The CFQ-R and CFQoL subscales illustrated significant associations with treatment descriptors and had strong correlation between each other. However, when the distribution of the three treatment burden instruments was assessed, the CFQoL illustrated a wider distribution across the sample compared to the other two instruments (ranging between 0 to 100). Despite the variation between the MTBQ and the two CF-specific subscales, all three instruments were associated with each other. The generic MTBQ illustrated moderate strength correlation with both subscales. This indicates that the MTBQ could be capturing some similar aspects of treatment burden to that measured by the two subscales.

The MTBQ is a generic measure of treatment burden that was developed for patients with multimorbidity. Despite CF being a multimorbidity condition, pwCF may not commonly relate to some of its items. For example, some items ask about the financial impact caused by treatment expenses ^12^; however, the majority of the sample did not have to pay for treatments. Some MTBQ items focus on the administrative aspect of treatment burden (monitoring health, arranging appointments, and collecting prescribed medications) and others on lifestyle changes caused by the disease (dieting and exercising) ^18^. These items could have captured perceived treatment burden based on the mentioned aspects; however, this cannot be confirmed since none of these aspects were used to assess the performance of the treatment burden instruments in this study. The other CF-specific subscales focus on treatment time and difficulty, in addition, to the psychological impact of the treatment i.e. life happiness. The variables used to describe treatments in this study (the number of treatments, treatment time, and complexity) capture some of the concepts in the CF-specific subscales, but not the generic MTBQ. Therefore, it is not surprising to find an association between the two CF-specific subscales and those variables. Moreover, the MTBQ was originally developed on a population older than the average age range of a person with CF (and the average age of this sample); hence, the generic MTBQ may lack face validity when it is used in pwCF.

The lack of correlation between perceived treatment burden and, age, gender and disease severity correspond with Sawicki *et al*. ^3^ findings. In their study, they only used the CFQ-R subscale to capture treatment burden, while this study used the CFQ-R in addition to the CFQoL subscale and the generic MTBQ. Moreover, we observed no difference in treatment burden between disease severity groups based on lung function, which agrees with Sawicki *et al*. ^3^ observation. These findings indicate that in CF, perceived treatment burden might be independent of age, gender, or the level of disease severity and cannot be predicted by them. This is perhaps not surprising as CF standards of care include a relatively strict regimen of therapies across the disease spectrum.

We found moderate to weak correlations between treatment burden, measured by CFQ-R, CFQoL subscales, and MTBQ and the number of daily treatments (the number of inhaled therapies, nebulized therapies, and chest physiotherapies). These findings correspond with Sawicki *et al*. ^3^ as they also found an association between treatment burden measured via CFQ-R and the number of nebulized therapies and chest physiotherapies. Based on these moderate to weak correlations, we assumed that the number of daily treatments might not have heavily impacted perceived treatment burden for pwCF as we expected. A person with multiple treatments may have less treatment burden if the treatments are easy to administer and fast to take than a person with fewer treatments but harder to administer and require longer time to take. Therefore, we assessed treatment complexity and treatment time.

The inverse correlations between perceived treatment burden measured by the CFQ-R and CFQoL subscales and the TCS were moderate in strength. The Sawicki *et al*. ^7^ study also showed negative correlation between treatment burden measured by CFQ-R and the TCS, however, the correlation was weak. Nonetheless, our findings do not imply that TCS fully capture the concepts covered by both treatment burden subscales. It is important to remember that TCS is an objective measure of complexity; while perceived treatment burden is based on how pwCF view their treatment and this may vary between different individuals.

Compared to the Sawicki *et al*. ^3^ study, our sample reported lower total treatment time in minutes per day (80 vs. 108 min). Also, we found moderate to weak associations between treatment burden measured by CFQ-R and CFQoL subscales and total treatment time which resembles Sawicki *et al*.’s ^3^ observations. These associations were anticipated since both CFQ-R and CFQoL subscales have items that ask about time spent in completing treatments.

Our study is the first to apply a generic measure of perceived treatment burden in a CF population and to compare the performance of different treatment burden instruments in CF. Nonetheless, this study had several limitations. First, the study included CF adults from one CF center which limits the generalizability of its results. Moreover, the cross-sectional nature of the study makes it hard to distinguish the confounding variables which makes it difficult to clearly interpret the results of the study. Further longitudinal studies are needed to confirm these outcomes and the ability of these instruments to capture changes of treatment burden over time. Some variables like treatment time were collected from the survey which is based on the participants memory and that can potentially introduce recollection bias. Also, the sample size in the overall study and across the disease severity groups were small due to the low survey response rate. This might have contributed in the lack of statistical significance of some of the observations, in particular the lack of variation between all the treatment burden instruments when compared across the disease severity groups.

## Conclusions

Treatment burden is considered a substantial problem for the CF population. Until now, the CFQ-R and CFQoL subscales are the only available measures to capture their perceived treatment burden. They both illustrated that treatment burden increases with more treatments, longer treatment time and more complex treatments. The generic MTBQ measure was not developed on a CF population and had almost no association with the treatment descriptors but showed correlations with the CFQ-R and CFQoL subscales. This is the first study to compare the performance of different treatment burden measures in CF adults, adding important insights into this high priority field. Further studies on this topic are needed, particularly if treatment recommendations change in the era of CFTR modulators. Qualitative studies that clearly describe the perceived treatment burden will be vital to capture these important issues as the health status and treatment options in CF continue to evolve. 

## Data availability

### Underlying data

The study sample was recruited from people attending the Adult Cystic Fibrosis Center at the Royal Brompton Hospital, linked demographic and clinical data were obtained from the UK CF Registry. Participants’ consent was associated only with the VALU-CF study, and not future studies. Additional permission must be sought from the hospital to use this data in further research. To request access to the full raw data, please contact Dr. Siobhan B. Carr, S.Carr@rbht.nhs.uk. Extensive summary data can be found in the article.

### Extended data

Figshare: Extended data for ‘Exploring the nature of perceived treatment burden: a study to compare treatment burden measures in adults with cystic fibrosis’. https://doi.org/10.6084/m9.figshare.19538560 ^14^.

Data are available under the terms of the Creative Commons Zero “No rights reserved” data waiver (CC0 Public domain dedication). 
