# Supplementary material for: Exploring the nature of perceived treatment burden: a study to compare treatment burden measures in adults with cystic fibrosis
Source: NIHR Open Res. 2022 May 9;2:36. [Version 1] doi: 10.3310/nihropenres.13260.1 (PMC7614250; doi:10.3310/nihropenres.13260.1)
Supplement: Peer review history

# Reviewer report: Reviewer response for version 1

**DOI:** 10.3310/nihropenres.14376.r28841

**Published:** 2022-11-10

**Authors:** Nataliya Kashirskaya

**Affiliations:** 1 Research Centre for Medical Genetics, Moscow, Russian Federation

Treatment burden in CF is substantial and multifactorial and its complexity increases dramatically with age. High treatment complexity tends to undermine quality of life and adherence to these treatments. In light of the recent progress toward normalizing the health of PwCF by restoring CFTR activity with a use of CFTR modulators, the need for symptomatic treatments that have been recommended for decades is being questioned in the last few years (chest physiotherapy, mucolytics, bronchodilators, nebulized antibiotics and macrolides, etc.). As it is mentioned by Altabee R. *et al* the only currently available instruments to capture perceived treatment burden for pwCF are the treatment burden subscales of the CF-specific quality of life measures; the CF questionnaire revised (CFQ-R) and the CF quality of life (CFQoL) instruments. Both subscales cover some concepts of treatment burden such as time spent on treatment, difficulties caused by the treatments, and interference with life and happiness, but don’t cover, for example, the burden of financial and managerial requirements associated with treatment. That’s why the authors decided to use an extra measurement - a generic treatment burden instrument - the Multimorbidity Treatment Burden Questionnaire (MTBQ) developed for a UK population. This generic instrument has not been evaluated in PwCF.

 This single survey study targeted 101 adults with CF in a large UK adult center. Participants completed an online survey that contained three different treatment burden measures; two short (three questions each) CF-specific questionnaires, and one generic (13 questions) questionnaire that captures treatment burden in people with long-term conditions. The study aimed to quantify perceived treatment burden in CF adults and to explore the relationship between perceived treatment burden and disease severity. This study also aimed to assess the performance of the three treatment burden instruments (CFQ-R, CFQoL, MTBQ) and their correlation with each other. The study was well designed and the data was statistically correctly processed. I have no major suggestions for revision or improvement of the study. The manuscript is very well illustrated.

 A high degree of treatment burden was reported by pwCF, as measured by the CFQ-R and CFQoL. The MTBQ showed slightly lower level of treatment burden compared to the other two subscales. There was no association observed between perceived treatment burden and the demographic and clinical variables; in addition to no significant difference in treatment burden between the disease severity groups categorized by lung function.

 The CFQoL illustrated a wider distribution across the sample compared to the other two instruments. Despite the variation between the MTBQ and the two CF-specific subscales, all three instruments were associated with each other. The generic MTBQ illustrated moderate strength correlation with both subscales. This indicates that the MTBQ could be capturing some similar aspects of treatment burden to that measured by the two subscales and can be used in future research in pwCF. This is the first study to compare the performance of different treatment burden measures in CF adults, adding important insights into this high priority field. By including treatment burden evaluation in the process of treatment decision-making is important as it takes into perspective the CF patient’s point of view about their treatment plan.

 I read with interest the comments by Reviewer #1 (Latchford G), Hoo ZH and Wildman M, and the response to them by Rana Altabee on behalf of the authors of the manuscript. Further studies on this topic are needed taking in mind that treatment recommendations will be definitely changing in the era of CFTR modulators.

Is the work clearly and accurately presented and does it cite the current literature?

Yes

If applicable, is the statistical analysis and its interpretation appropriate?

Yes

Are all the source data underlying the results available to ensure full reproducibility?

Yes

Is the study design appropriate and is the work technically sound?

Yes

Are the conclusions drawn adequately supported by the results?

Yes

Are sufficient details of methods and analysis provided to allow replication by others?

Yes

Reviewer Expertise:

Cystic fibrosis

I confirm that I have read this submission and believe that I have an appropriate level of expertise to confirm that it is of an acceptable scientific standard.

# Response

**Published:** 2022-11-14

**Authors:** Rana Altabee

**Affiliations:** University of East Anglia, UK

We thank the reviewer for her helpful and valuable feedback.

 Currently, the CF-specific quality of life measures (CFQ-R and CFQoL) treatment burden subscales are the only available methods used to capture treatment burden from the pwCF perspective. However, treatment burden is much bigger in concept than what’s covered by the 3-itemed subscales. Therefore, we hope with this study to highlight the importance of treatment burden for the CF population, especially with the availability of the CFTR modulators.

# Reviewer report: Reviewer response for version 1

**DOI:** 10.3310/nihropenres.14376.r28651

**Published:** 2022-06-01

**Authors:** Gary Latchford

**Affiliations:** 1 Leeds Institute of Health Sciences, University of Leeds, Leeds, UK

This study focusses on a very important area within CF - treatment burden. As they say, it was ranked the top research question by the James Lind Alliance.

 It's an important but quite tricky concept - as they note, it means different things to different people. This paper focused on measurement, and was a well conducted, very sensible piece of research. I have no major suggestions for revision or improvement.

 Using a cross sectional sample of 101 participants from one large UK CF centre in London, they compared the performance of the very short treatment burden subscales from two QoL measures (CFQ-R and CFQoL), and a generic treatment burden measure (MTBQ). They also very wisely used Sawicki's treatment complexity score to allow for some interesting analysis.

 The findings are pretty intuitive, but very useful to see properly explored. All the measures correlate with each other and all showed a higher treatment burden to be associated with more treatments. The CFQ-R and CFQol both performed better with CF patients however, demonstrating an association between longer treatment times and more complex treatments with a higher treatment burden that was not present in the MTBQ data. This makes sense. Many of the items on the MTBQ do not translate well onto the treatment context associated with CF. They note this and I'd go further - one of the key features of CF treatment is that it is delivered via a dedicated, accessible team in a specialist centre normally very well known to the person with CF. I expect this minimises a lot of the practical challenges other patients may face. 

 It's interesting that severity is not associated with treatment burden. They note that this most likely reflects the considerable treatment regimen all are asked to follow. I wonder too whether it reflects the ongoing, day to day routine of this burden - I expect when asked about treatment burden people will tend to recall a generic rather than specific memory.

 Finally, I agree that this is a particularly important area just now, with the potential in the future for modulator therapies to mean other treatments may no longer be needed. It's important to track this change. The two CF QoL measures seem adequate, but that might change as the nature of the treatment in CF changes. I wondered if there were any thoughts about developing a new measure? 

 Data availability - consent was only for the current study but the data may be available with permission from the hospital.

Is the work clearly and accurately presented and does it cite the current literature?

Yes

If applicable, is the statistical analysis and its interpretation appropriate?

Yes

Are all the source data underlying the results available to ensure full reproducibility?

Partly

Is the study design appropriate and is the work technically sound?

Yes

Are the conclusions drawn adequately supported by the results?

Yes

Are sufficient details of methods and analysis provided to allow replication by others?

Yes

Reviewer Expertise:

Clinical Health Psychology; Motivational interviewing; cystic fibrosis

I confirm that I have read this submission and believe that I have an appropriate level of expertise to confirm that it is of an acceptable scientific standard.

# Response

**Published:** 2022-06-08

**Authors:** Rana Altabee

**Affiliations:** University of East Anglia, UK

We thank the reviewer for his kind and helpful comments.

 We hope that this study would highlight the importance of capturing treatment burden in people with CF, especially with the new era of modulators. Since the CF-specific treatment burden subscales were the most adequate in capturing treatment burden in CF, it would be great to see future studies developing a new treatment burden instrument for CF.
